# Chewing Gum: Cognitive Performance, Mood, Well-Being, and Associated Physiology

**DOI:** 10.1155/2015/654806

**Published:** 2015-05-17

**Authors:** Andrew P. Allen, Andrew P. Smith

**Affiliations:** ^1^Laboratory of Neurogastroenterology, Department of Psychiatry/Alimentary Pharmabiotic Centre, Biosciences Institute, University College Cork, Cork, Ireland; ^2^Centre for Occupational and Health Psychology, School of Psychology, Cardiff University, 63 Park Place, Cardiff CF10 3AS, UK

## Abstract

Recent evidence has indicated that chewing gum can enhance attention, as well as promoting well-being and work performance. Four studies (two experiments and two intervention studies) examined the robustness of and mechanisms for these effects. Study 1 investigated the acute effect of gum on mood in the absence of task performance. Study 2 examined the effect of rate and force of chewing on mood and attention performance. Study 3 assessed the effects of chewing gum during one working day on well-being and performance, as well as postwork mood and cognitive performance. In Study 4, performance and well-being were reported throughout the workday and at the end of the day, and heart rate and cortisol were measured. Under experimental conditions, gum was associated with higher alertness regardless of whether performance tasks were completed and altered sustained attention. Rate of chewing and subjective force of chewing did not alter mood but had some limited effects on attention. Chewing gum during the workday was associated with higher productivity and fewer cognitive problems, raised cortisol levels in the morning, and did not affect heart rate. The results emphasise that chewing gum can attenuate reductions in alertness, suggesting that chewing gum enhances worker performance.

## 1. Introduction

Chewing gum can enhance alertness and sustained attention, although its effects upon stress may differ depending upon whether chronic or acute stress is examined; see reviews by Allen and Smith [1] and Hirano and Onozuka [2]. Chewing gum has enhanced sustained attention performance in previous research [3, 4], consistent with an alerting effect of chewing gum [4–6]. There is some evidence that this effect may be moderated by time-on-task, with the ameliorating effect of gum being greater following a long period of performance [6, 7]. Neuropsychological data further confirms an enhancement of sustained attention by gum. The event related potential P300, which is associated with vigilance, had a shortened latency following chewing gum [8], and frontal and temporal beta power were heightened by chewing gum following performance of a sustained attention task [9]. Quantitative EEG effects of chewing gum without cognitive performance seem to be moderated by flavour [10, 11], suggesting that alertness may be altered by chewing gum in the absence of cognitive performance. Quickening of reaction time on an adapted version of the attention network task [12] was associated with increased activity in motor regions for alerting and executive networks, as well as the anterior cingulate cortex and left frontal gyrus for the executive network [13]. Hirano et al. demonstrated this effect using gum without flavour or odour, suggesting that the motor activity of chewing may be a key factor in explaining these results; however, it remains unclear if a greater level of motor activity in chewing will heighten any associated effects. Although there is evidence that more vigorous chewing or greater resistance to chewing does not moderate chewing effects on memory [14, 15], the fact that chewing gum can enhance arousal which is depleted by attention tasks (e.g., by heightening heart rate and beta power during vigilance) [9] suggests that it is more plausible that more vigorous chewing could have a greater effect on attention.

Consistent with an alerting effect of chewing gum under laboratory conditions, chewing gum during the workday has also been shown to enhance self-reported productivity both in university staff [16] and in university students [17], consistent with an improvement in sustained attention. Although chewing gum has been associated with increased heart rate in experimental studies [9, 18] it remains unclear if sympathetic nervous system arousal may explain enhanced performance in an everyday working context.

People who chew gum habitually report less stress [19, 20], and chewing gum has reduced anxiety [21] and reported stress [22] induced by an acute social stressor, although other studies have not found a reduction on acute stress or anxiety [23, 24]. If chewing gum can reduce feelings of stress it may attenuate feelings of depression, a stress-related disorder. Strikingly, in a clinical sample of mild-moderately depressed patients, depression was reduced to a greater extent when gum was administered with antidepressant medication, compared to medication alone [25]. In a nonclinical sample, chewing gum for two weeks can reduce feelings of stress, anxiety, and depression in university staff [16], as well as reducing stress in university students [17]. In summary, it would appear that there is clearer evidence for an ameliorating effect of gum on chronic stress compared to acute stress [1]. Given this contrast between short- and long-term effects, it remains unclear if a shorter intervention (one day) can reduce feelings of stress, anxiety, and depression in a sample of working adults.

The current research aims to examine the effect of gum on well-being and cognitive performance by combining the study of chewing effects under controlled conditions with a more naturalistic examination of chewing gum during the workday. We firstly examined the acute effect of chewing gum on mood in the absence of cognitive performance (Study 1: Mood Effects in the Absence of Performance). Although previous research on mood effects of gum has examined chewing in the absence of cognitive performance, this has been in the context of sleep deprivation [26] or neurological testing, rather than under less demanding conditions. We then assessed the effects of intensity of chewing on mood and cognitive performance (Study 2: Rate of Chewing, Mood, and Cognition). To examine subjective and performance effects of chewing gum on an ongoing basis in a naturalistic setting we then tested the effects of chewing gum on well-being and performance during a single workday, to examine if effects observed over longer intervention periods are robust enough to be demonstrated within this time frame (Study 3: Working Day Intervention: Well-Being and Performance). The final study again examined a single workday intervention (Study 4: Working Day Intervention: Well-Being, Performance, and Physiology); underlying physiological mechanisms for effects on well-being and performance, which have previously been studied only under more acute testing conditions, were probed by examining changes in salivary cortisol and heart rate over the course of the working day while chewing gum.

## 2. Study 1: Mood Effects in the Absence of Performance

### 2.1. Methods

All studies described in this paper received ethical approval from Cardiff University's School of Psychology Ethics Committee and were conducted in accordance with the Declaration of Helsinki.

#### 2.1.1. Participants

One hundred adults (81 females, 19 males; mean age = 21.1, SD = 3.6) were recruited. Participants were mostly students from the School of Psychology, Cardiff University. For all studies, people taking medication, who reported medical problems, who consumed more than 40 units of alcohol per week, or who smoked more than 10 cigarettes in the daytime and evening, were excluded from participation. Participants were recruited through a university notice board and an online experiment management system.

#### 2.1.2. Materials

*Chewing Gum*. Wrigley's extra spearmint and Wrigley's gum base (synthetic rubber) were provided.

*Mood Task*. The mood task was presented on a desktop PC. Participants completed the tasks using a purpose-built response box with three large square buttons (“A” on the left, “B” on the right, and “Space” in the centre). Mood was measured using 18 bipolar visual analogue scales or VAS. Scores for alertness (maximum score = 400), hedonic tone (maximum score = 300), and anxiety (maximum score = 150) were derived from these scales. The component scales for alertness were drowsy/alert, strong/feeble, coordinated/clumsy, attentive/dreamy, lethargic/energetic, muzzy/clear headed, incompetent/proficient, and mentally slow/quick witted. The scales for hedonic tone were contented/discontented, happy/sad, antagonistic/friendly, interested/bored, self-centred/outward going, and withdrawn/sociable. The scales for anxiety were relaxed/excited, troubled/tranquil, and tense/calm. There was no time limit for this task. This mood scale has previously shown sensitivity to changes in mood in response to chewing gum [6].

#### 2.1.3. Design

Participants were assigned at random to one of four conditions: chewing spearmint gum with replacement of gum (female = 20, male = 5), chewing gum without replacement (female = 22, male = 3), chewing gum base (female = 21, male = 4), and no chewing (female = 18, male = 6).

#### 2.1.4. Procedure

Testing was scheduled for between 10.00 and 12.00. Participants filled in questionnaires assessing demographic information and habitual gum consumption on arrival. They were then provided with two pieces of spearmint gum or gum base if they were in a chewing condition and told to chew constantly throughout the procedure. Immediately after starting to chew gum they completed the initial mood assessment tasks. They were then requested to sit quietly and continue chewing. After 15 minutes, participants in a chewing condition were verbally reminded to continue chewing, and those in the replacement condition were reminded to replace the gum with two new pellets if the current gum had lost its flavour. Psychology textbooks and journals were available for participants to read, and participants could bring their own reading material. After 25 minutes, the participants filled in the final mood assessment task.

#### 2.1.5. Statistical Analysis

This analysis was conducted in two stages, with the first stage testing the effect of chewing gum per se, by comparing the no-gum control to the three gum conditions combined, using 2 × 2 mixed ANOVA, with the independent variables being time (initial and final assessment) and chewing (chewing versus no chewing). The second stage evaluated differences between all four gum conditions, using 2 × 4 mixed ANOVA, with the independent variable being time (as above) and gum condition (spearmint with replacement, spearmint gum without replacement, gum base, and no-gum control). The dependent variables were alertness, hedonic tone, and anxiety.

### 2.2. Results

#### 2.2.1. The Effect of Time on Mood

Alertness fell significantly between the initial and final assessment, *F*(1,96) = 24.17, *P* < .001, and* partial η* ^2^ = .2. Anxiety rose between the initial and final measurement, although this effect was only marginally significant, *F*(1,94) = 3.57, *P* = .06, and* partial η* ^2^ = .04. Hedonic tone fell significantly over the course of the study, *F*(1,96) = 29.15, *P* < .001, and* partial η* ^2^ = .23. Time had a significant effect on all components of hedonic tone, except self-centred/outward going.

#### 2.2.2. The Effect of Chewing Gum on Mood

**Figure 1:**
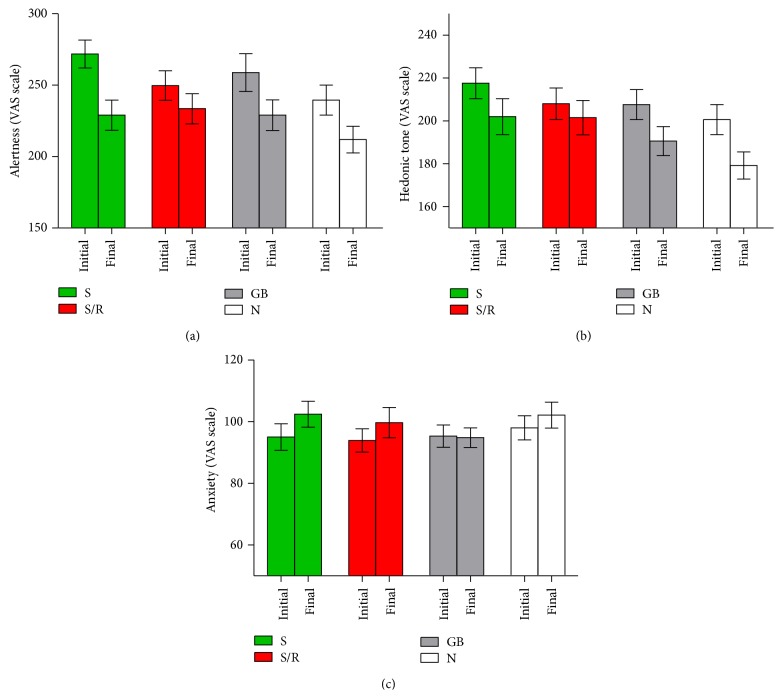
Chewing gum and initial and final mood (Study 1). (a) Alertness. (b) Hedonic tone. (c) Anxiety (S = spearmint gum without replacement, S/R = spearmint gum with replacement, GB = gum base, and N = no-gum control). Error bars represent standard error of the mean.

Averaging across gum conditions, alertness was higher in chewing gum conditions compared to the control, *F*(1,98) = 3.92, *P* = .05, and* partial η* ^2^ = .04, but gum did not moderate the change in alertness between initial and final alertness, *F*(1,98)<.001, *P* = .99, and* partial η* ^2^ < .001. Although alertness fell by slightly less in the gum with replacement condition, gum flavour and replacement did not significantly moderate changes in alertness between the initial and final assessment of mood, *F*(3,96) = .59, *P* = .62, and* partial η* ^2^ = .02, nor did flavour and replacement have a significant main effect on alertness, *F*(3,96) = 1.61, *P* = .19, and* partial η* ^2^ = .05 (see Figure 1(a)).

Comparing the no-gum control to all gum conditions, there was a trend for gum to increase hedonic tone, *F*(1,98) = 3.54, *P* = .06, and* partial η* ^2^ = .04, although chewing gum did not moderate the difference between final and initial hedonic tone, *F*(1,98) = 1.68, *P* = .2, and* partial η* ^2^ = .02. Although hedonic tone fell somewhat less in the gum with replacement condition, there was no significant effect of gum condition on change in hedonic tone, *F*(3,96) = 1.25, *P* = .3, and* partial η* ^2^ = .04 or main effect of gum condition on hedonic tone, *F*(3,96) = 1.59, *P* = .2, and* partial η* ^2^ = .05 (see Figure 1(b)).

Comparing the no-gum control to all gum conditions, gum did not have a main effect on anxiety, *F*(1,98) = .6, *P* = .44, and* partial η* ^2^ = .006, and there was no interaction between chewing gum and time, *F*(1,98) = 3.54, *P* = .99, and* partial η* ^2^ < .001. The gum conditions did not have a main effect on anxiety, *F*(3,96) = .37, *P* = .78, and* partial η* ^2^ = .01, nor was there a significant effect of gum condition on change in anxiety over time, *F*(3,96) = .86, *P* = .47, and* partial η* ^2^ = .03 (see Figure 1(c)).

### 2.3. Study 1 Discussion

Consistent with multiple studies examining chewing gum during cognitive performance, the results of Study 1 indicate that chewing gum may increase alertness in the absence of cognitive performance tasks. There was also a trend for hedonic tone to be increased by chewing gum. However, in the absence of cognitive performance tasks anxiety was not affected by chewing gum. The observed alerting effect was not dependent upon mint flavor; it may be the case that chewing plays a key role in such an alerting effect. It is thus of interest if the rate of chewing may moderate alerting effects of gum.

## 3. Study 2: Rate of Chewing, Mood, and Cognition

This experiment examined if rate of chewing could potentially moderate the effects of gum on attention and mood. Participants were filmed while chewing in order to establish the rate of chewing (pilot data indicated good interrater reliability for scoring of number of chews per minute).

### 3.1. Methods

#### 3.1.1. Participants

Fifty-six adults (42 females, 14 males; mean age = 19.6, SD = 1.4) were recruited. Participants were mostly students from the School of Psychology, Cardiff University.

#### 3.1.2. Materials

*Chewing Gum*. As a moderating effect of flavour was not observed in Study 1, participants were given a choice of flavours for this study, as well as Studies 3 and 4. The following chewing gums were available: Wrigley's spearmint, Wrigley's extra (flavours: spearmint, peppermint, cool breeze, and ice), and Wrigley's airwaves (flavours: cherry, green mint, black mint, menthol, and eucalyptus).

*Cognitive Tasks*

*Selective Attention Tasks [27]*

*(i) Focused Attention Task*. In this task target letters appeared as upper case A's and B's in the centre of the screen. Participants were required to identify as quickly and as accurately as possible if the target letter was an A or a B, by pressing A or B with the forefinger of the left or right hand, while ignoring any distracters presented elsewhere on the screen. Before each presentation of the target, three warning crosses were displayed for 500 ms. The middle cross was then replaced by the target, and the outer crosses were replaced by distracters (in the case of trials with distracters). The outer crosses were separated from the middle cross by 1.02° or 2.6°. The target letter was accompanied by nothing, letters which were the same as the target, letters which were different from the target, or asterisks.

Mean reaction time, number of errors, and number of long responses (>800 ms) were measured. The threshold for long responses was based on previous research [28]. Breadth of attention was also assessed (the difference in reaction time and accuracy between targets with distracters presented near to the target versus targets with distracters at a further distance from the target). The difference in reaction time between conditions where the target changed from the previous trial and where it remained the same was used as a measure of speed of encoding of new information. Following 10 practice trials, participants completed three blocks of 64 trials. This test lasted approximately 5 minutes.

*(ii) Categoric Search Task*. This task was similar to the focused attention task previously outlined, including number of practice and experimental trials. However, in this task participants did not know where the target would appear. At the start of each trial, two crosses appeared 2.04° or 5.2° apart or further apart, located towards the left or right extremes of the display. The target then replaced one of these crosses. For half the trials the target was presented alone and for half it was accompanied by a distracter (a digit from 1 to 7).

Mean reaction time, accuracy, and long responses (>1000 ms) were recorded, as well as reaction time and accuracy with which new information was encoded. Differences in reaction time and accuracy for trials where the position of the target stimulus and response key were compatible versus where they were incompatible were used as a measure of response organisation. The effect of the stimulus appearing in a different location versus the same location as the previous trial was measured, as well as the effect of not knowing the location of the target. This task also lasted approximately 5 minutes.

*Variable Fore-Period Simple Reaction Time Task [29]*. In this task a box was displayed on the screen, followed by a square being presented in the middle of the box. The participant had to press the “Space” button as soon as the square was detected. The period of time elapsed before each appearance of the square varied. This task lasted 3 minutes.

*Repeated Digits Vigilance Task [29]*. Three-digit numbers were shown on the screen at the rate of 100 per minute. Each number was normally different from the preceding one, but for 8 occasions per minute the number presented was the same as that presented on the previous trial. Participants had to detect these repetitions and respond by hitting the “Space” button as quickly as possible. The number of hits (correctly detected repetitions), reaction time for hits, and number of false alarms were recorded. The task lasted 5 minutes.

#### 3.1.3. Design

Each participant completed both the chewing gum and no-gum control conditions. Similar to previous studies, gum condition was included as a crossover variable to test if any effects of gum would carry over to a no-gum condition (for those who completed the gum condition first).

#### 3.1.4. Procedure

Following informed consent and a familiarisation with the mood and attention tasks, participants completed the mood and attention tasks twice. Participants were instructed to chew two pieces of gum constantly at their own pace during one of these testing sessions and not to chew during the other testing session. Each set of the mood and attention tasks took approximately 25 minutes, and participants completed the second condition immediately after the first. Participants selected a packet of gum just before the chewing condition. They were filmed throughout the chewing session. In order to assess the rate of chewing during each task, notes were taken of when each computerised task began and ended. This timing of the tasks was matched to the footage of the participant completing the task, so that the rate of chewing during each specific task could be calculated. Participants indicated how hard they had been chewing on a scale of 1 (as softly as possible) to 11 (as hard as possible) immediately after the gum condition.

#### 3.1.5. Analysis

*Analysis of Footage*. The footage was divided into the mood tasks, blocks for the selective attention tasks, and minutes for the simple reaction time task and repeated digits vigilance task, as well as gaps between tasks. Each piece of footage was rated twice, and the intraclass correlation (single measures) was .996, suggesting excellent test/retest reliability for the video rating. The mean of the two scores for each section of the footage was used as the final result.

*Statistical Analysis*. Mixed ANOVA was used to assess the effect of chewing gum (repeated measures: gum versus no-gum control), order of gum condition (independent measures: gum condition first versus gum condition second), and time-on-task. Time-on-task was entered as a repeated measures variable in the analysis of variables for which time-on-task data was available (i.e., alertness, hedonic tone, and anxiety, categoric search reaction time, focused attention reaction time, simple reaction time, repeated digits hit, false alarms, and reaction time). Time-on-task was defined as pre- versus posttest for reported mood (i.e., before and after the attention tasks) and blocks or minutes for cognitive tasks.

Multiple regressions with forced entry were used to test if the predictors were associated with changes in attention and mood between gum and no-gum conditions. The predictors were rate of chewing, speed of chewing and intensity (how hard gum was chewed), and prior amount of chewing (total count of times chewed; this did not apply for pretest mood, when chewing had just begun).

### 3.2. Results

#### 3.2.1. Chewing Gum and Mood

**Figure 2:**
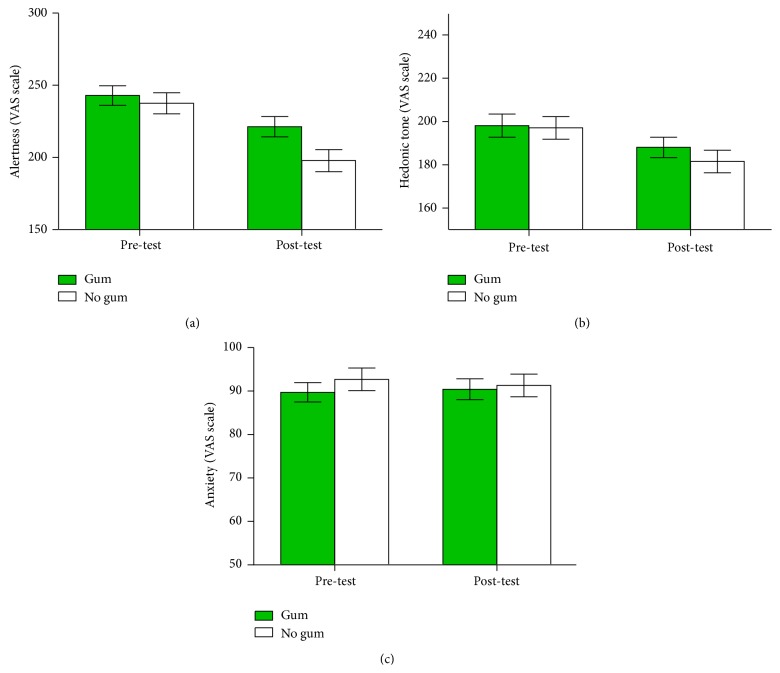
Chewing gum, pre- and posttest mood (Study 2). (a) Alertness. (b) Hedonic tone. (c) Anxiety. Error bars indicate standard error of the mean.

There was a significant main effect of time and chewing gum on alertness; alertness fell between pre- and posttest assessments, *F*(1,54) = 57.13, *P* < .001, and* partial η* ^2^ = .51, and chewing gum was associated with higher alertness, *F*(1,54) = 24.62, *P* < .001, and* partial η* ^2^ = .31. There was also an interaction between gum condition and time, *F*(1,54) = 8.47, *P* = .005, and* partial η* ^2^ = .14; alertness was higher in the gum condition posttest. There was a significant interaction between gum and order of gum condition, *F*(1,54) = 11.5, *P* = .001, and* partial η* ^2^ = .18. Alertness was improved to a greater extent by chewing gum when it came first (see Figure 2(a)).

Hedonic tone fell significantly between pre- and posttest, *F*(1,54) = 62.45, *P* < .001, and* partial η* ^2^ = .54, and hedonic tone was significantly higher in the gum condition, *F*(1,54) = 6.74, *P* = .01, and* partial η* ^2^ = .11, but there was not a significant interaction between gum and time, *F*(1,54) = 2.32, *P* = .13, and* partial η* ^2^ = .04. There was a significant interaction between gum and order of gum condition, *F*(1,54) = 14.43, *P* < .001, and* partial η* ^2^ = .21. Hedonic tone was improved to a greater extent by chewing gum when it came first (see Figure 2(b)).

There was no significant effect of time on anxiety, *F*(1,54) = .09, *P* = .77, and* partial η* ^2^ = .002, nor was there a significant main effect of chewing gum, *F*(1,54) = 2.75, *P* = .1, and* partial η* ^2^ = .05. There was no interaction between gum and time, *F*(1,54) = 1.4, *P* = .24, and* partial η* ^2^ = .03, and there was no interaction between gum and order of gum condition, *F*(1,54) = .76, *P* = .39, and* partial η* ^2^ = .01 (see Figure 2(c)).

#### 3.2.2. Chewing Gum, Time-on-Task, and Cognition

**Figure 3:**
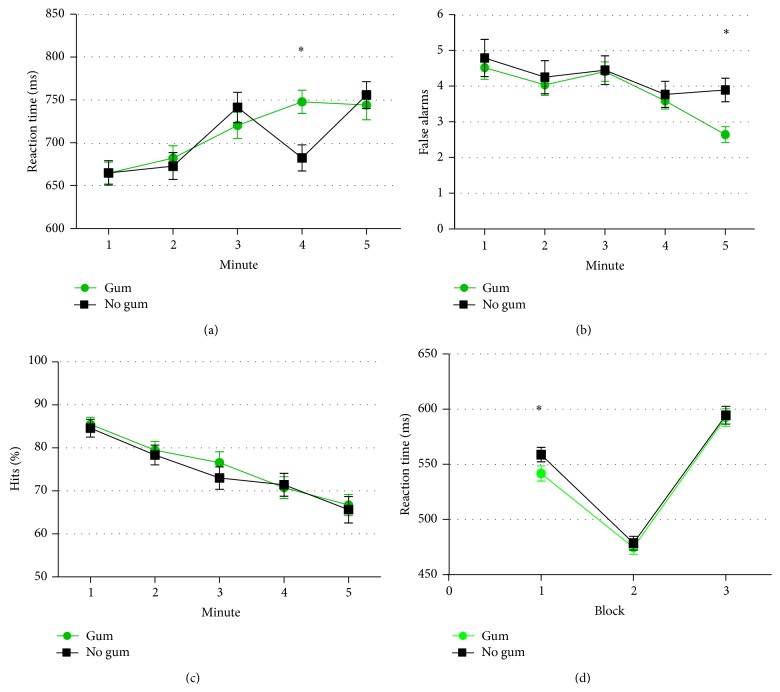
Time-on-task trends in chewing gum effects on (a) vigilance reaction time, (b) vigilance false alarms, (c) vigilance hits, and (d) categoric search reaction time (Study 2). Error bars represent standard error of the mean.

Chewing gum had a significant main effect on categoric search speed of encoding. There was a significant interaction between gum condition and time-on-task for repeated digits reaction time, *F*(4,216) = 4.22, *P* = .003, and* partial η* ^2^ = .07 (see Figure 3(a)). Chewing gum lengthened reaction time during the fourth minute, *F*(1,54) = 13.91, *P* < .001, and* partial η* ^2^ = .21, indicating a negative effect of chewing gum on performance at this time. There was also a main effect of time, *F*(4,216) = 20.53, *P* < .001, and* partial η* ^2^ = .28, with reaction time lengthening over time, but there was not a main effect of chewing gum, *F*(1,54) = 1.04, *P* = .31, and* partial η* ^2^ = .02. There was no significant interaction between gum and order of gum condition, *F*(1,54) = .04, *P* = .85, and* partial η* ^2^ = .001.

There was a gum by time-on-task interaction for false alarms, *F*(4,216) = 2.25, *P* = .048, and* partial η* ^2^ = .05; chewing gum reduced the number of false alarms during the final minute of the task, *F*(1,54) = 13.69, *P* = .001, and* partial η* ^2^ = .2 (see Figure 3(b)), indicating a positive effect on performance during the final minute. There was a significant main effect of time-on-task, *F*(4,216) = 9.07, *P* < .001, and* partial η* ^2^ = .14, with the number of false alarms falling during later minutes. There was, however, no main effect of chewing gum on false alarms, *F*(1,55) = 1.52, *P* = .22, and* partial η* ^2^ = .03. There was an interaction between gum and order of gum condition *F*(1,54) = 6.7, *P* = .01, and* partial η* ^2^ = .11. False alarms were heightened by gum when gum came before the no-gum control.

For vigilance hits, there was no significant main effect of chewing gum, *F*(1,54) = .91, *P* = .35, and* partial η* ^2^ = .02 or interaction between gum and time-on-task, *F*(4,216) = .28, *P* = .89, and* partial η* ^2^ = .005 (see Figure 3(c)). Again, there was a main effect of time-on-task, with percent hits falling during later minutes, *F*(4,216) = 31.27, *P* < .001, and* partial η* ^2^ = .37. There was an interaction between gum and order of gum condition, *F*(1,54) = 16.5, *P* < .001, and* partial η* ^2^ = .23. Hits were enhanced by chewing gum when it came before the no-gum control.

There was a gum × time interaction for categoric search reaction time, with gum shortening reaction time, but only during the first block, *F*(2,108) = 5.76, *P* = .004, and* partial η* ^2^ = .1 (see Figure 3(d)). This was in the context of a strong main effect of time, *F*(2,108) = 5.92, *P* = .004, and* partial η* ^2^ = .1, with reaction time significantly shortened during the second block, although there was not a main effect of gum, *F*(1,55) = .01, *P* = .95, and* partial η* ^2^ < .001.

Gum had a significant main effect on focused attention speed of encoding, with slower encoding of information in the gum condition. There was a significant interaction between chewing gum and order of gum condition for focused attention mean reaction time, errors, speed of encoding, and simple reaction time. For simple reaction time, performance was improved by gum when it came after the control condition, while the opposite was true for focused attention. Results are summarised in Table 1.

#### 3.2.3.  Rate of Chewing, Mood, and Cognition

A faster rate of chewing was associated with lengthened simple reaction time (beta = .42, *P* = .04). Harder chewing was associated with faster encoding of new information on the categoric search task (beta = −.37, *P* = .02). Greater prior chewing was associated with a higher level of focused attention errors (beta = .32, *P* = .04). Rate of chewing, force of chewing, and prior chewing did not moderate mood or performance on the repeated digits vigilance task. Results are summarised in Table 2.

### 3.3. Study 2 Discussion

Consistent with previous research as well as Study 1, chewing gum was associated with higher alertness. This might be expected to improve sustained attention performance, although the results indicated lengthened reaction time as well as fewer false alarms as the vigilance task continued, suggesting negative and positive effects on sustained attention performance. Vigilance performance was not moderated by rate of chewing; however, although faster chewing was associated with lengthened simple reaction time, harder chewing was associated with faster encoding of new information on the categoric search task, and prior chewing was associated with more errors on the focused attention task. It thus may be useful for researchers to take some measure of how hard and fast participants are chewing in future research.

In order to further examine the effects of chewing gum on performance and reported feelings in a more naturalistic setting, the next studies examined chewing gum over the course of a working day.

## 4. Study 3: Working Day Intervention: Well-Being and Performance

This study examined the effects of chewing gum over a single workday on reported well-being and performance. We hypothesised that chewing gum would be associated with improved well-being and performance at work.

### 4.1. Method

#### 4.1.1. Participants

One hundred and twenty-six adults (87 females, 39 males) were recruited. Mean age was 29 (SD = 6.7). Participants were full-time university staff; their occupations were administration/secretary (*N* = 36), researcher/lecturer (36), management (12), technician (10), applied psychologist (4), marketing (4), support worker (4), dentist (2), teacher (2), and other occupations indicated by one participant each (16).

#### 4.1.2. Materials

Chewing gum was the same as used in Study 2.

*Well-Being and Performance at Work*. Self-report questionnaires were used to assess well-being and performance at work. The Hospital Anxiety and Depression Scale (HADS; [30]) was used; based on principal component analysis of survey data [31], we divided outcomes into inattention/hyperactivity (composed of the items “I feel restless as if I have to be the move,” “I have lost interest in my appearance,” and “I can enjoy a good book or radio or TV programme”) as well as the anxiety and depression, based on the remaining items from these original categories. The fatigue subscale from the profile of fatigue-related symptoms (PFRS; [32]) was used to assess fatigue, as well as a single-item question on how stressful participants found their job (as opposed to life in general). Single-item questions were also used to assess occupational performance; these questioned participants on cognitive failures and productivity/being behind with work (on scales from 0 to 4). These measures had all been used by Smith et al. [16].

#### 4.1.3. Design

The study comprised a one-day intervention; participants were randomly assigned to a chewing condition (female = 39, male = 23) or nonchewing condition (female = 48, male = 16).

#### 4.1.4. Procedure

During an initial study visit before the main testing day, participants completed a familiarisation with the tasks performed on the PC and completed a questionnaire concerning general levels of well-being and performance at work (these acted as baseline scores of well-being and performance). Participants also provided information about demographics, occupation, and habitual level of chewing gum. On the testing day, participants completed a full battery of the mood and attention tasks in the morning as baseline measures. They were required to chew gum (one full packet of 10 pieces) or avoid chewing gum over the course of the working day. Participants were informed that they could chew when they wished during the working day, although they were encouraged to chew when they felt stressed, and they were told to eat and drink as much as they usually would. They returned to the laboratory following work and completed the same well-being questionnaire as in the familiarisation, except this time pertaining to how they felt that workday. They then completed the full battery again, to assess the effects of gum chewing during the workday; no one chewed gum during this battery.

#### 4.1.5. Statistical Analysis

Analyses of covariance were used, with chewing gum condition as the predictor, baseline scores as covariates, and well-being and performance as dependent variables.

### 4.2. Results

Chewing gum was associated with reduced occupational stress, *F*(1,119) = 3.83, *P* = .027, and* partial η* ^2^ = .03, inattention/hyperactivity, *F*(1,118) = 3.0, *P* = .04, and* partial η* ^2^ = .03, and fatigue, *F*(1,123) = 3.57, *P* = .03, and* partial η* ^2^ = .03. Anxiety was slightly higher in the chewing gum group, as was depression, although these differences were nonsignificant (see Table 3). During the one-day intervention, chewing gum was significantly associated with reporting of fewer cognitive problems, *F*(1,122) = 7.18, *P* = .008, and* partial η* ^2^ = .06 and lower levels of being behind with work, *F*(1,122) = 5.5, *P* = .02, and* partial η* ^2^ = .04.

### 4.3. Study 3 Discussion

The results indicated that, similar to a previous intervention using the same measures but lasting for two weeks [16], chewing gum for a single working day was associated with lower job stress, fatigue, and inattention. The findings of improved reported performance, as well as the reduction in fatigue and inattention, chime with the findings of heightened alertness from Studies 1 and 2. However, after adjustment for baseline differences, anxiety and depression were not higher in the chewing gum condition. It is of interest if a physiological mechanism might underpin these findings. Consequently, in Study 4 heart rate and cortisol were measured to examine if these would also be altered by chewing gum over the course of a working day.

## 5. Study 4: Working Day Intervention: Well-Being, Performance, and Physiology

In this study we examined the effects of chewing gum on well-being and performance as well as heart rate and cortisol over the course of a working day. We hypothesised that chewing gum would reduce cortisol, consistent with a reduction in stress, and increase heart rate, consistent with findings of improved performance.

### 5.1. Methods

#### 5.1.1. Participants

These were thirty full-time university staff (23 females, 7 males). Mean age was 30.4 (SD = 6.9). Their occupations were administration/secretary (*N* = 12), researcher (9), and other occupations indicated by only one participant each (9).

#### 5.1.2. Materials

Chewing gum as well as mood and well-being measures was the same as those used in Studies 2 and 3. Heart rate was measured using Polar s610 heart rate monitors with Spectra 360 gel. Saliva samples were collected using Sarstedt salivettes.

#### 5.1.3. Design

Participants completed both chewing and no-gum control conditions in a crossover design.

#### 5.1.4. Procedure

During a familiarisation day, participants spent a workday wearing a heart rate monitor, giving saliva samples and recording well-being and performance at the same time as they did during the main testing days. The main testing took place over two separate days. Chewing gum was consumed during one testing day and avoided during the other, control day. The testing days were at least one week apart, in order to avoid carryover effects. Participants came into the lab before work (between 8 a.m. and 9.30 a.m.) to collect heart rate monitors, salivettes, gum (in the gum condition), and questionnaires (if using hard copies).

Participants were requested to chew a full packet of gum during the intervention day. Participants were emailed online links or given hard copies of questionnaires, which were filled in at 10.00, 11.00, 12.00, 14.00, and 15.00. Participants were free to chew gum before filling in the first questionnaire at 10.00. Saliva samples were taken at the same time as the questionnaires. Heart rate was measured throughout the working day.

Participants were requested not to eat for one hour before the postwork session. After work, well-being and performance were assessed again. Participants were instructed to keep saliva samples refrigerated after being taken. Saliva samples were frozen in a −20 freezer on return to the laboratory.

#### 5.1.5. Analysis

*Physiological Analysis*. Cortisol levels were measured in duplicate by radioimmunoassay adapted from Read et al. [33]. The limit of detection was .7 nmol/L, intra-assay coefficient of variation was 10.8%, 8.8%, and 5.3% at 3.3, 6.4, and 24.7 nmol/L, respectively, and interassay variation was 11.0%, 10.8%, and 10.7% at 2.5, 5.1, and 26.4 nmol/L. Heart rate data was visually examined for artefacts and these were removed.

*Statistical Analysis*. The effects of gum (gum versus no gum) and time of day (10.00, 11.00, 12.00, 14.00, and 15.00) were analysed using repeated measures 2 × 5 ANOVA. The effect of gum as reported at the end of the day was analysed using repeated measures *t*-tests. As the testing days were separated by at least one week, order of gum condition was not entered into the analysis.

### 5.2. Results

#### 5.2.1. Chewing Gum, Performance, and Well-Being

**Figure 4:**
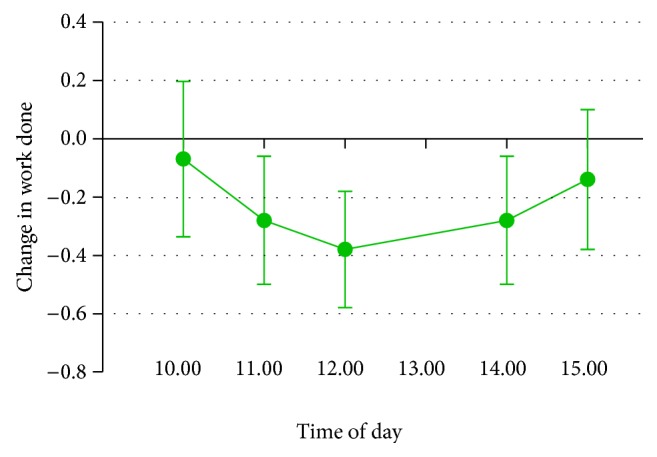
Change between gum conditions in work done (being behind with work) during working day (Study 4). Lower difference scores indicate higher productivity in the gum condition compared to no-gum control. Error bars represent standard error of the mean.

There was a trend for work done reported during the day to be higher in the gum condition, *F*(1,23) = 3.28, *P* = .08, and* partial η* ^2^ = .13, with participants reporting being less behind with work (see Figure 4). There were no other effects of gum on well-being or performance during the workday (see Table 4). There were no significant interactions between gum condition and time of day for well-being and performance.

At the end of the workday, reporting of cognitive problems was lower in the gum condition than in the control. The gum intervention reduced anxiety and inattention/hyperactivity reported at the end of the day, although these effects were not significant. The effects of chewing gum reported at the end of the intervention conditions are summarised in Table 5.

#### 5.2.2. Chewing Gum and Physiology

**Figure 5:**
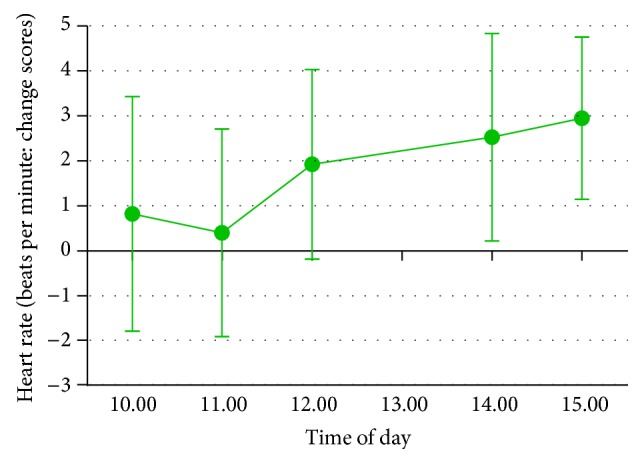
Change between gum conditions in heart rate over course of working day (Study 4). Higher difference scores indicate higher heart rate in the gum condition compared to no-gum control. Error bars represent standard error of the mean.

*Heart Rate*. Heart rate was higher during the gum condition for both regular chewers, *M* = 1.6 (change in beats per minute), SD = 8.8, and nonregular chewers, *M* = .8, SD = 5.9. There was a significant main effect of time of day, with heart rate at its lowest between 10 and 12, *F*(4,92) = 21.94, *P* < .001, and* partial η* ^2^ = .49. However, there was no significant main effect of gum, *F*(1,23) = .87, *P* = .36, and* partial η* ^2^ = .04, nor was there an interaction between gum and time, *F*(4,92) = .29, *P* = .88, and* partial η* ^2^ = .01 (see Figure 5).

**Figure 6:**
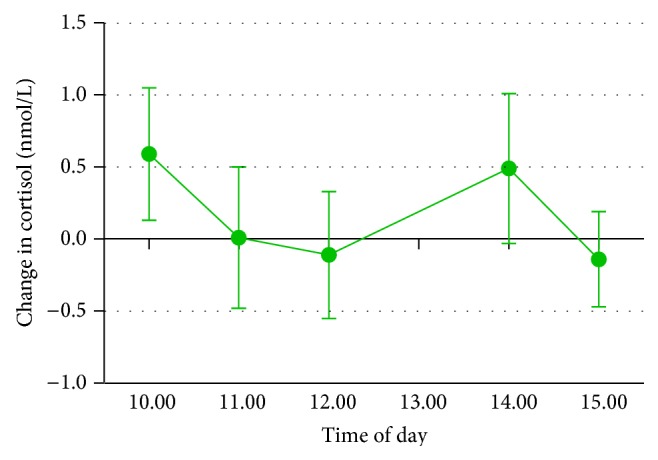
Change between gum conditions in cortisol over course of working day (Study 4). Error bars represent standard error of the mean.

*Cortisol*. The interaction between gum condition and time of day was nonsignificant overall, *F*(2.97,65.3) = .82, *P* = .24, and* partial η* ^2^ = .04 (Greenhouse-Geisser adjusted). However, salivary cortisol was higher in the gum condition for the first testing period at 10 a.m., *F*(1,25) = 332.46, *P* < .001, and* partial* eta squared = .91 (see Figure 6).

### 5.3. Study 4 Discussion

Similar to Study 3, chewing gum was associated with reduced reporting of cognitive problems, along with a trend for being less behind with work, although we did not observe a positive effect on fatigue, inattention, or job stress in the current study, suggesting that over a one-day intervention the effect of chewing gum is more robust for performance than for well-being. There was some preliminary evidence that cortisol was increased in the morning, although heart rate was not significantly enhanced by chewing gum.

## 6. General Discussion

This research offers further evidence in support of an alerting effect of chewing gum, which was associated with heightened alertness both with and without cognitive performance. Although it is a possibility that those in chewing gum condition were coincidentally in a more alert state on entering the lab (which would be captured by a baseline mood measure) and that this carried over to the initial mood rating, there is previous evidence that chewing gum is associated with improved mood rated just after receiving gum [4, 34]. Chewing gum was also associated with reduced fatigue during a working day in Study 3 (Working Day Intervention: Stress and Performance), although this was not replicated within participants in Study 4 (Working Day Intervention: Stress, Performance, and Physiology). Neither rate of chewing nor flavour of gum moderated the alerting effect, suggesting the effect is not dependent upon mint flavour or intensity of chewing.

Under experimental conditions in Study 2, chewing gum had varying effects on sustained attention performance, with lengthened reaction time in the fourth minute but fewer false alarms during the final minute. This is consistent with previous evidence, which has suggested generally positive but sometimes mixed effects of gum on attention [2]. Although the reduction in false alarms suggests a positive performance effect and slowing in reaction time suggests a negative effect, both findings are consistent with the trade-off between speed and accuracy in vigilance performance. In the findings of Tucha et al. [35], chewing gum had a negative impact on the performance of a vigilance task for children with ADHD as well as normal children. They observed lengthened reaction time in the gum condition, similar to the present findings. However, children with ADHD made more omission errors (in the current research hits and consequently omission errors were not affected by gum) and neither group of children were affected in terms of commission errors (in contrast to the current research, which found a positive impact in terms of reduced false alarms later in the task). There is relatively limited research on chewing gum effects on sustained attention in children, but these findings suggest that children may respond in different ways to chewing gum and specifically that it may not have a beneficial effect in the context of ADHD. Faster chewing was associated with lengthened simple reaction times; a possible explanation for this is that of distraction, as suggested previously [36].

Chewing gum during the working day was associated with reduced cognitive problems and enhanced productivity in both Study 3 and Study 4, suggesting that the experimental findings on sustained attention may generalise to the working environment. Similar to the experimental work of Smith [4] as well as that of Gray et al. [22], there was some preliminary evidence that cortisol was enhanced following chewing gum. However, this was only the case during the initial stages of the day, suggesting that cortisol secretion is not increased throughout the day by chewing gum. The cortisol results also contrast with those of Scholey et al. [37], who observed a decrease in cortisol. This decrease in cortisol may be due to the fact that they used a different stressor compared to Gray et al., who used a psychosocial stress procedure, and Study 4 of the current research, which examined naturalistic cortisol changes over the working day. Although an increase in heart rate and improved vigilance have been observed experimentally following chewing gum [9], heart rate was not affected by chewing gum during the workday, suggesting sympathetic arousal may only be relevant for short-term effects of chewing gum. Previous research which indicated that chewing gum increased heart rate has also found that it improved sustained attention [9] and aspects of memory [18]; this could be an area of interest for further research.

Chewing gum reduced stress in Study 3, but not in Study 4, and in contrast to previous research, such as that of Smith et al. [16], chewing gum did not affect anxiety and depression. This may be due to the relatively brief duration of the chewing gum intervention compared to previous research, which typically employed two weeks of chewing gum. Within the depressed sample in Erbay et al. [25], chewing gum was clearly associated with alterations in gastrointestinal symptoms, suggesting that chewing gum may have a beneficial role in the brain-gut axis [38]; it may thus be of interest if chewing gum can ameliorate gastrointestinal symptoms in stress-related brain-gut axis disorders such as irritable bowel syndrome, although it should be noted that irritable bowel syndrome is associated with a prolonged cortisol response to acute stress [39], so chewing gum may not be beneficial in irritable bowel syndrome under stressful conditions if it increases cortisol.

There are a number of other different mechanisms which could explain the observed effects of chewing gum, such as facial muscle activation [4], as EMG has been shown to be maintained when sustained attention performance declines less [40]. However, as a greater rate of chewing would require greater activation of facial muscles, it seems unlikely that facial muscle activation is impacting on sustained attention in a dose-response manner given the current findings. Another mechanism could be altered central nervous system activity [9, 13, 41–43], perhaps due to a stimulation of regional blood flow or glucose delivery [44]. Allen et al. found enhanced beta activity with flavourless chewing gum; this is consistent with the finding from Study 1 that flavour did not appear to moderate any alerting effect of chewing gum. However, although Allen et al. observed increased heart rate under acute experimental conditions, the current results do not provide evidence for increased heart rate over the course of the working day. It should be borne in mind that, as chewing gum had a rapid effect on mood in Study 1, there should be a mechanism which is rapid-acting that could explain these effects.

Similar to a number of previous studies on the effects of chewing gum on cognition and mood [23, 37], we used predominantly female samples. There is previous survey evidence that females are more likely to chew gum than males [19], so it is likely that this is representative of broader consumption patterns.

Future research in this area could assess the psychophysiological effects of chewing gum in more depth; indices of heart rate variability and ambulatory blood pressure have been associated with work stress [45] and so may be informative of effects of chewing gum on stress. Given clearer effects of chewing gum during longer interventions it may be the case that cortisol secretion may be reduced along with occupational stress after two weeks of chewing gum. It would be useful to assess level of physical activity during the workday in future research; experimental conditions may be associated with a consistent level of physical activity, but activity levels may differ substantially between individuals' working days. This would lead to higher variability in heart rate, in which gum has been shown to increase under controlled, low-activity conditions [9, 18]. Physical activity can also impact upon cortisol levels [46, 47]; closer monitoring of physical activity requiring participants to avoid intense physical activity before and during a study could help to obtain more reliable results.

## 7. Conclusions

Chewing gum was associated with enhanced productivity and reduced cognitive errors at work, as well as heightened cortisol in the morning. However, rate of chewing, flavor, or cognitive performance did not moderate the enhancement of alertness and changes in sustained attention by chewing gum, suggesting that greater motor activity does not exaggerate these effects.

## Figures and Tables

**Table 1:** Chewing gum, time-on-task, and attention.

	Gum	No gum	Results
Focused attention			
Mean reaction time (ms)	Block 1: *M* = 396.14 (5.89) Block 2: *M* = 395.2 (5.38) Block 3: *M* = 400.18 (5.31)	Block 1: *M* = 391.97 (5.85) Block 2: *M* = 397.55 (5.26) Block 3: *M* = 401.39 (5.23)	Gum: *F*(1, 54) = .01, *P* = .94, *η*_*p*_^2^ < .001 Time^**^: *F*(2, 108) = 5.82, *P* = .004, *η*_*p*_^2^ = .1 Gum × time: *F*(1.802, 99.12) = 1.44, *P* = .24, *η*_*p*_^2^ = .03 Gum × order: *F*(1, 54) = 3.89, *P* = .054, *η*_*p*_^2^ = .06
Total errors	10.18 (1.12)	10.15 (1.04)	Gum: *F*(1, 54) = .003, *P* = .96, *η*_*p*_^2^ < .0001 Gum × order^††^: *F*(1, 54) = 5.37, *P* = .02, *η*_*p*_^2^ = .09
Long responses	.27 (.09)	.45 (.21)	Gum: *F*(1, 54) = .91, *P* = .35, *η*_*p*_^2^ = .02 Gum × order: *F*(1, 54) = 2.94, *P* = .09, *η*_*p*_^2^ < .05
Breadth of attention^1^	18.99 (4.71)	25.83 (5.39)	Gum: *F*(1, 54) = 1.03, *P* = .32, *η*_*p*_^2^ = .02 Gum × order: *F*(1, 54) = .78, *P* = .38, *η*_*p*_^2^ = .01
Speed of encoding^2^	25.47 (2.77)	24.44 (2.61)	Gum: *F*(1, 54) = .21, *P* = .64, *η*_*p*_^2^ = .004 Gum × order^††^: *F*(1, 54) = 12.15, *P* < .001, *η*_*p*_^2^ = .18

Categoric search			
Total errors	11.16 (.8)	11.84 (.93)	Gum: *F*(1, 54) = 1.45, *P* = .23, *η*_*p*_^2^ = .03 Gum × order: *F*(1, 54) = 2.51, *P* = .12, *η*_*p*_^2^ = .04
Long responses	1.66 (.3)	1.87 (.36)	Gum: *F*(1, 54) = .45, *P* = .5 *η*_*p*_^2^ = .008 Gum × order: *F*(1, 54) = .83, *P* = .37, *η*_*p*_^2^ = .02
Response organisation^3^	27.51 (2.54)	26.88 (2.53)	Gum: *F*(1, 54) = .05, *P* = .083 *η*_*p*_^2^ = .001 Gum × order: *F*(1, 54) = .07, *P* = .79, *η*_*p*_^2^ = .001
Speed of encoding	17.69 (2.73)	4.77 (2.54)	Gum^††^: *F*(1, 54) = 14.3, *P* < .001, *η*_*p*_^2^ = .21 Gum × order: *F*(1, 54) = .04, *P* = .84, *η*_*p*_^2^ = .001
Spatial uncertainty^4^	105.92 (4.83)	116.26 (5.34)	Gum: *F*(1, 54) = 3.28, *P* = .08, *η*_*p*_^2^ = .06 Gum × order: *F*(1, 54) = .043, *P* = .52, *η*_*p*_^2^ = .008
Place repetition^5^	15.62 (2.56)	14 (2.92)	Gum: *F*(1, 54) = .35, *P* = .56, *η*_*p*_^2^ = .006 Gum × order: *F*(1, 54) = .72, *P* = .4, *η*_*p*_^2^ = .01

Simple reaction time	Block 1: *M* = 327.98 (6.39) Block 2: *M* = 336.58 (7.14) Block 3: *M* = 339.29 (6.68)	Block 1: *M* = 314.43 (7.32) Block 2: *M* = 331.02 (7.44) Block 3: *M* = 341.09 (8)	Gum: *F*(1, 54) = 2.04, *P* = .16, *η*_*p*_^2^ = .04 Time^††^: *F*(2, 108) = 16.09, *P* < .001, *η*_*p*_^2^ = .23 Gum × time: *F*(2, 108) = 2.07, *P* = .13, *η*_*p*_^2^ = .04 Gum × order^***^: *F*(1, 54) = 22.08, *P* = .001, *η*_*p*_^2^ = .29

Standard errors of the means are in parentheses. ^1^Higher score = broader focus of attention. ^2^Higher score = slower encoding of information. ^3^Higher score = poorer organisation. ^4^Higher score = greater uncertainty. ^5^Higher score = greater effect of place repetition. ^**^indicates *P* < .01, ^††^indicates *P* < .001, and ^***^indicates *P* = .001. Gum × gum order refers to interaction between gum condition and order in which gum condition appeared.

**Table 2:** Level of chewing and its effect on mood and cognition.

	Unstandardised *B*	SE *B*	Beta	Significance	*R* ^2^	Adjusted *R* ^2^
Mood						
Pretest alertness					.03	−.01
Constant	26.45	18.14		.15		
Rate of chewing	−.57	.62	−.13	.36		
Intensity	−1.04	2.65	−.06	.7		
Posttest alertness					.06	.01
Constant	20.74	13.08		.12		
Rate of chewing	−.9	.6	−.32	.14		
Prior chewing	.01	.02	.12	.61		
Intensity	2.19	2.53	.13	.39		
Pretest hedonic tone					.02	−.02
Constant	.56	9.77		.95		
Rate of chewing	−.25	.33	−.11	.45		
Intensity	1.44	1.43	.15	.32		
Posttest hedonic tone					.04	−.02
Constant	11.67	7.37		.12		
Rate of chewing	−.41	.34	−.26	.23		
Prior chewing	.004	.01	.11	.47		
Intensity	−.33	1.43	−.04	.82		
Pretest anxiety					.05	.01
Constant	−9.62	−.01		.11		
Rate of chewing	−.006	.2	−.01	.98		
Intensity	1.31	.85	.22	.13		
Posttest anxiety					.02	−.04
Constant	.2	4.07		.96		
Rate of chewing	−.09	.19	−.11	.62		
Prior chewing	−.001	.005	−.04	.86		
Intensity	.29	.79	.06	.71		
Focused attention						
Mean reaction time (ms)					.02	−.04
Constant	.8	8.93		.93		
Rate of chewing	.25	.29	.14	.39		
Prior chewing	−.003	.01	−.04	.81		
Intensity	−1.01	1.78	−.09	.57		
Total errors					.1	.05
Constant	−1.6	2.09		.45		
Rate of chewing	.02	.07	.04	.81		
Prior chewing^*^	.01	.003	.32	.04		
Intensity	−.15	.42	−.05	.72		
Number of long responses					.02	−.03
Constant	.1	.58		.86		
Rate of chewing	.01	.02	.04	.81		
Prior chewing	.001	.001	.12	.46		
Intensity	−.11	.12	−.15	.34		
Breadth of attention					.11	.06
Constant	−47.15	19.44		.02		
Rate of chewing	1.01	.63	.27	.09		
Prior chewing	−.02	.03	−.12	.46		
Intensity	4.3	3.87	.17	.27		
Speed of encoding					.03	−.03
Constant	−6.53	7.38		.38		
Rate of chewing	.13	.24	.09	.58		
Prior chewing	.003	.01	.04	.79		
Intensity	.69	1.47	.07	.64		
Categoric search						
Mean reaction time					.01	−.05
Constant	−1.86	12.14		.88		
Rate of chewing	.01	.44	.002	.99		
Prior chewing	.004	.02	.04	.83		
Intensity	−1.49	2.42	−.1	.54		
Total errors					.03	−.03
Constant	−2.12	1.72		.23		
Rate of chewing	−.01	.06	−.04	.83		
Prior chewing	−.001	.002	−.07	.72		
Intensity	.42	.34	.19	.23		
Long responses					.11	.06
Constant	1.84	.88		.04		
Rate of chewing	−.04	.03	−.22	.2		
Prior chewing	<.001	.001	.06	.73		
Intensity	−.26	.18	−.23	.14		
Response organization					.01	−.05
Constant	1.43	8.79		.87		
Rate of chewing	.21	.32	.12	.52		
Prior chewing	−.003	.01	−.05	.8		
Intensity	−.7	1.75	−.06	.69		
Speed of encoding					.11	.05
Constant	29.75	9.82		.004		
Rate of chewing	−.03	.36	−.02	.93		
Prior chewing	.02	.01	.21	.24		
Intensity^*^	−4.74	1.96	−.37	.02		
Spatial uncertainty					.05	−.004
Constant	−15.94	16.94		.35		
Rate of chewing	−.88	.61	−.25	.16		
Prior chewing	.01	.02	.09	.63		
Intensity	3.56	3.38	.16	.3		
Place repetition					.02	−.03
Constant	−2.41	8.23		.77		
Rate of chewing	−.1	.3	−.06	.73		
Prior chewing	.01	.01	.18	.35		
Intensity	.09	1.64	.009	.95		
Simple reaction time					.08	.03
Constant	−21.09	15.47		.18		
Rate of chewing^*^	.84	.49	.42	.04		
Prior chewing	.007	.02	.05	.7		
Intensity	.74	2.69	.04	.79		
Repeated digits vigilance						
Percent hits					.09	.04
Constant	1.41	1.79		.44		
Rate of chewing	−.11	.05	−.32	.06		
Prior chewing	<.001	.002	−.03	.86		
Intensity	.27	.33	.12	.42		
False alarms					.04	−.01
Constant	−.73	5.31		.89		
Rate of chewing	.17	.16	.17	.31		
Prior chewing	.002	.006	.06	.69		
Intensity	−1.11	.98	−.18	.26		
Reaction time					.06	.007
Constant	−17.49	24.72		.48		
Rate of chewing	−1.2	.75	−.27	.12		
Prior chewing	.02	.03	.12	.41		
Intensity	6.89	4.56	.23	.14		

^*^Indicates *P* < .05.

**Table 3:** Well-being and performance at baseline and following one-day chewing gum intervention/no-gum control.

	Baseline		Intervention	
	Chewing gum	No gum	Chewing gum	No gum
Job stress	1.44 (.1)	1.48 (.07)	1.08 (.12)^*^	1.42 (.11)
Fatigue	2.39 (.12)	2.26 (.11)	2.18 (.14)^*^	2.33 (.12)
Anxiety	5.08 (.35)	4.63 (.29)	3.03 (.3)	2.61 (.29)
Depression	2.72 (.28)	2.12 (.24)	2.42 (.28)	1.97 (.23)
Inattention	2.17 (.17)	2.32 (.18)	2.05 (.2)^*^	2.52 (.21)
Behind with work	2.31 (.1)	2.48 (.11)	1.35 (.13)^**^	1.84 (.13)
Cognitive problems	1.97 (.12)	1.98 (.11)	1.01 (.11)^†^	1.39 (.12)

Standard errors of the means are in parentheses. Significant effects of gum intervention compared to no-gum, adjusting for baseline scores: ^*^indicates *P* < .05, ^†^indicates *P* = .01, and ^**^indicates *P* < .01.

**Table 4:** Mean change between gum and control conditions in well-being and performance during the workday.

	10 a.m.	11 a.m.	12 noon	2 p.m.	3 p.m.	Results
Cognitive problems	−.03 (.13)	−.03 (.18)	−.27 (.2)	−.11 (.17)	−.41 (.22)	Gum: *F*(1, 23) = 1.03, *P* = .32, *η*_*p*_^2^ = .04 Time^*^: *F*(2.68, 61.61) = 3.95, *P* = .02, *η*_*p*_^2^ = .15 Gum × time: *F*(4, 92) = .41, *P* = .8, *η*_*p*_^2^ = .02

Job stress	0 (.25)	−.07 (.25)	−.17 (.17)	−.32 (.21)	−.24 (.18)	Gum: *F*(1, 24) = .67, *P* = .42, *η*_*p*_^2^ = .03 Time^†^: *F*(2.82, 67.71) = 3.46, *P* = .01, *η*_*p*_^2^ = .13 Gum × time: *F*(2.01, 48.3) = .27, *P* = .77, *η*_*p*_^2^ = .01

Fatigue	−.16 (.32)	−.24 (.36)	−.25 (.39)	−.9 (.4)	−.81 (.45)	Gum: *F*(1, 25) = 2.99, *P* = .1, *η*_*p*_^2^ = .11 Time: *F*(2.08, 51.99) = 1.05, *P* = .36, *η*_*p*_^2^ = .04 Gum × time: *F*(4, 100) = .86, *P* = .49, *η*_*p*_^2^ = .03

Anxiety	0 (.21)	0 (.16)	−.4 (.17)	−.07 (.1)	.07 (.16)	Gum:* F*(1, 26) = .19, *P* = .67, *η*_*p*_^2^ = .007 Time^*^:* F*(4, 104) = 2.49, *P* = .047, *η*_*p*_^2^ = .09 Gum × time:* F*(2.74, 71.31) = .1.32, *P* = .28, *η*_*p*_^2^ = .05

Depression	.13 (.14)	.18 (.13)	−.03 (.14)	.07 (.09)	.14 (.15)	Gum: *F*(1, 22) = .01, *P* = .91, *η*_*p*_^2^ = .001 Time: *F*(2.2, 47.9) = 2.04, *P* = .14, *η*_*p*_^2^ = .09 Gum × time: *F*(4, 88) = 1.51, *P* = .21, *η*_*p*_^2^ = 06

Standard errors of the means are in parentheses. ^*^indicates *P* < .05; ^†^indicates *P* = .01.

**Table 5:** Mean change between gum and control conditions in well-being and performance reported at the end of the workday.

Behind with work	−.13 (.21)	*t*(29) = .54, *P* = .54, Cohen's *d* = .11
Cognitive problems^*^	−.35 (.15)	*t*(29) = −2.31, *P* = .03, Cohen's *d* = .42
Job stress	−.12 (.12)	*t*(29) = −.94, *P* = .35, Cohen's *d* = .17
Fatigue	.02 (.11)	*t*(29) = .21, *P* = .84, Cohen's *d* = .04
Anxiety	−.49 (.36)	*t*(29) = −1.38, *P* = .18, Cohen's *d* = .25
Depression	.25 (.35)	*t*(29) = .72, *P* = .48, Cohen's *d* = .13
Inattention	−.37 (.25)	*t*(29) = −1.48, *P* = .15, Cohen's *d* = .27

^*^Indicates significant effect of gum intervention, *P* < .05. Negative score indicates lower score in gum condition. Standard errors of the mean are in parentheses.
